# Cytotoxic effects of halophilic archaea metabolites on ovarian cancer cell lines

**DOI:** 10.1186/s12934-023-02206-y

**Published:** 2023-09-28

**Authors:** Magdalena Kowalewicz-Kulbat, Krzysztof T. Krawczyk, Izabela Szulc-Kielbik, Sebastian Rykowski, Marta Denel-Bobrowska, Agnieszka B. Olejniczak, Camille Locht, Magdalena Klink

**Affiliations:** 1 Department of Immunology and Infectious Biology, Institute of Microbiology, Biotechnology and Immunology, Faculty of Biology and Environmental Protection, University of Lodz, Lodz, Poland; 2 Institute of Medical Biology, Polish Academy of Sciences, Lodz, Poland; 3 Univ. Lille, CNRS, Inserm, CHU Lille, Institut Pasteur de Lille, U1019 – UMR9017 – CIIL – Center for Infection and Immunity of Lille, 59000 Lille, France

**Keywords:** Archaea, Halophiles, Ovarian cancer cells, Cytotoxicity

## Abstract

**Background:**

Ovarian cancer is one of the most frequent and deadly gynaecological cancers, often resistant to platinum-based chemotherapy, the current standard of care. Halophilic microorganisms have been shown to produce a large variety of metabolites, some of which show toxicity to various cancer cell lines. However, none have yet been shown to be active against ovarian cancer cells. Here, we examined the effects of metabolites secreted by the halophilic archaea *Halorhabdus rudnickae* and *Natrinema salaciae* on various cancer cell lines, including ovarian cancer cell lines.

**Results:**

^1^H NMR analyses of *Hrd. rudnickae* and *Nnm. salaciae* culture supernatants contain a complex mixture of metabolites that differ between species, and even between two different strains of the same species, such as *Hrd. rudnickae* strains 64^T^ and 66. By using the MTT and the xCELLigence RTCA assays, we found that the secreted metabolites of all three halophilic strains expressed cytotoxicity to the ovarian cancer cell lines, especially A2780, as well as its cisplatin-resistant derivative A2780cis, in a dose-dependent manner. The other tested cell lines A549, HepG2, SK-OV-3 and HeLa were only minimally, or not at all affected by the archaeal metabolites, and this was only seen with the MTT assay.

**Conclusions:**

The halophilic archaea *Hrd. rudnickae* and *Nnm. salaciae*, isolated from a Polish salt mine and Lake Medee in the Mediterranean Sea, respectively, secrete metabolites that are active against ovarian cancer cells, including those that are resistant to cisplatin. This opens potential new possibilities for the treatment of these frequent and deadly gynaecological cancers.

## Background

According to the World Health Organization (WHO), cancer was the first or second leading cause of death before the age of 70 years in 112 of 183 countries and ranked third or fourth in a further 23 countries in 2019 [1]. Ovarian cancer is the third most common gynaecologic malignancy worldwide and the leading cause of global gynaecologic oncology-related deaths [2]. More than 300,000 new cases of ovarian cancer are diagnosed and 18,000 patients die from their disease each year [3]. A major reason for the high mortality rate of ovarian cancer is the lack of characteristic symptoms leading to delayed diagnosis. For most patients (60–70%) ovarian cancer is diagnosed only at a very late stage III or IV, according to the International Federation of Gynecology and Obstetrics (FIGO) classification, which results in poor prognosis. The 5-year survival of patients with advanced disease is very low and is reached by only 25–35% of women [4, 5]. The second problem related to ovarian cancer is its high resistance to platinum-based chemotherapy. While the initial response may be promising, with 70–80% of the patients responding positively, depending on the stage of the disease, ovarian cancer recurs in up to 30% of patients at an early stage, to up to 85% of patients at an advanced stage within 6–24 months after chemotherapy due to acquired platinum resistance [6–8]. Therefore, there is an urgent need to develop new drugs with effective anti-tumor effects.

Halophilic microorganisms constitute natural microbial communities of hypersaline ecosystems, which are widely distributed around the world [9]. Their great metabolic diversity, low nutritional requirements and genetic machineries of adaptation to extreme conditions, such as high ionic strength, make them promising candidates as producers of anti-tumor metabolites. Culture supernatants of *Halobacterium salinarum* IBRC-M 10715 were found to contain metabolites with anti-cancer activity against prostate carcinoma and carotenoids isolated from *Halogeometricum limi* RO1-6, *Haloplanus vescus* RO5-8 and *Halobacterium halobium* strain M8 possess anti-cancer activity in liver hepatocellular adenocarcinoma [10]. However, the potential anti-cancer effects of metabolites produced by halophiles against ovarian cancer have not yet been described. Here, we examined the potential cytotoxic effect of metabolites secreted into the culture media of the halophilic archaea *Halorhabdus rudnickae* and *Natrinema salaciae* on various cancer cell lines (ovarian, lung, cervix and hepatocellular carcinomas) and found significant and specific cytotoxicity of these metabolites on the ovarian cancer cell lines A2780, A2780cis and SK-OV-3.

## Results

### ^1^H NMR analysis of culture supernatants of *Nnm. salaciae* DSM25055^T^, *Hrd. rudnickae* 64^T^ and *Hrd. rudnickae* 66

**Fig. 1.**
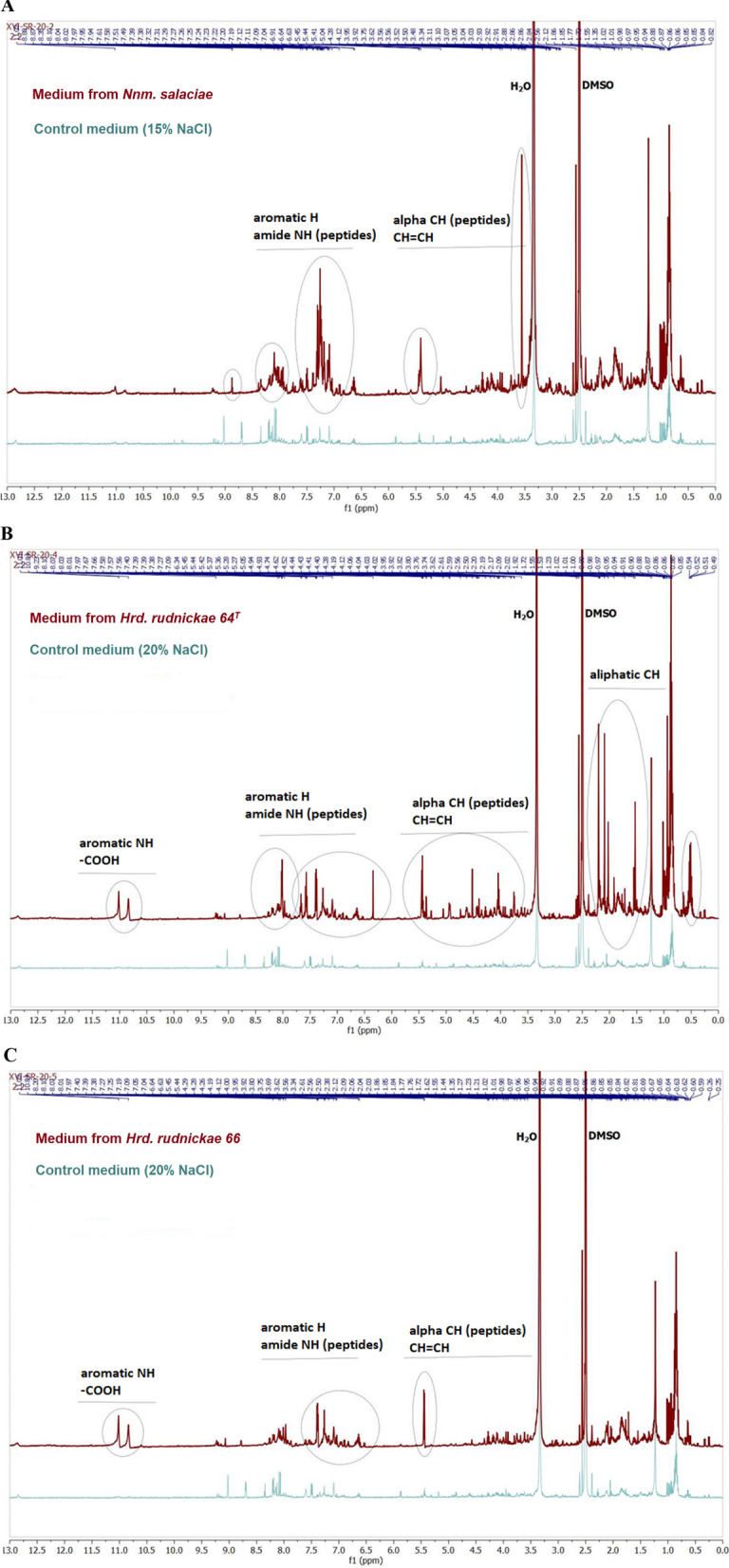
^1^H NMR spectrum of *Nnm. salaciae*, *Hrd. rudnickae* 64^T^ and *Hrd. rudnickae* 66 culture supernatants. *Nnm. salaciae* (**A**), *Hrd. rudnickae* 64^T^ (**B**) and *Hrd. rudnickae* 66 (**C**) were grown in HBM medium containing 15% (**A**) or 20% (**B** and **C**) NaCl for 48 h at 45 °C (**A**) or 37 °C (**B** and **C**). Supernatants were harvested by centrifugation, and ^1^H NMR spectra of the supernatant extracts were recorded (in red) and compared to the corresponding fresh media (in green). Significant peaks containing archaea-specific metabolites are circled

To determine whether halophilic archaea secrete compounds with potential anti-cancer activity, extracts of control media and media harvested after the growth of halophilic archaea were analyzed by ^1^H NMR spectroscopy. Compared to control media spectrograms, we have observed additional signals in spectra of media from cultures of *Nnm. salaciae* DSM25055^T^*, Hrd. rudnickae* 64^T^ and *Hrd. rudnickae* 66 (Fig. 1A–C).

According to their chemical shifts, the additional signals may come from compounds such as amino acids, peptides, nucleotides, nucleotides-related metabolites and metabolites associated with energy metabolism. Similar groups of compounds based on comparable chemical shifts of signals were observed in ^1^H NMR spectra of *Vibrio parahaemolyticus* [11]. Moreover, the spectra of the tested archaeal samples exhibited different complexities and intensities between strains. Interestingly, even the two strains of the same *Hrd. rudnickae* species differed in released metabolites. The *Hrd. rudnickae* 64^T^ sample contained higher amounts and showed more diversity of metabolites compared to the *Hrd. rudnickae *66 sample. As expected, the control spectrograms were identical regardless of the concentration of salt that was used in the media.

### Cytotoxicity of metabolites secreted by halophilic archaea

The cytotoxic activity of halophilic archaea samples was evaluated on various cell lines derived from different cancers. We used two assays to evaluate cytotoxicity, the MTT and the xCELLigence RTCA (real time cell analysis). The MTT assay is based on the reduction of tetrazolium salt to formazan through the mitochondrial oxidoreductase and dehydrogenase enzymes and electron donors, mainly NAD(P)H [12]. To complement the results obtained with the MTT assay, which measures metabolic activity of cells, we also used the xCELLigence RTCA, which measures real-time cell growth and viability. The CI (Cell Index) curves reveal information on the general behaviour of cells, such as growth and proliferation, changes in cell morphology, including size, volume and shape, and number of cells [13].

### MTT assay

**Fig. 2:**
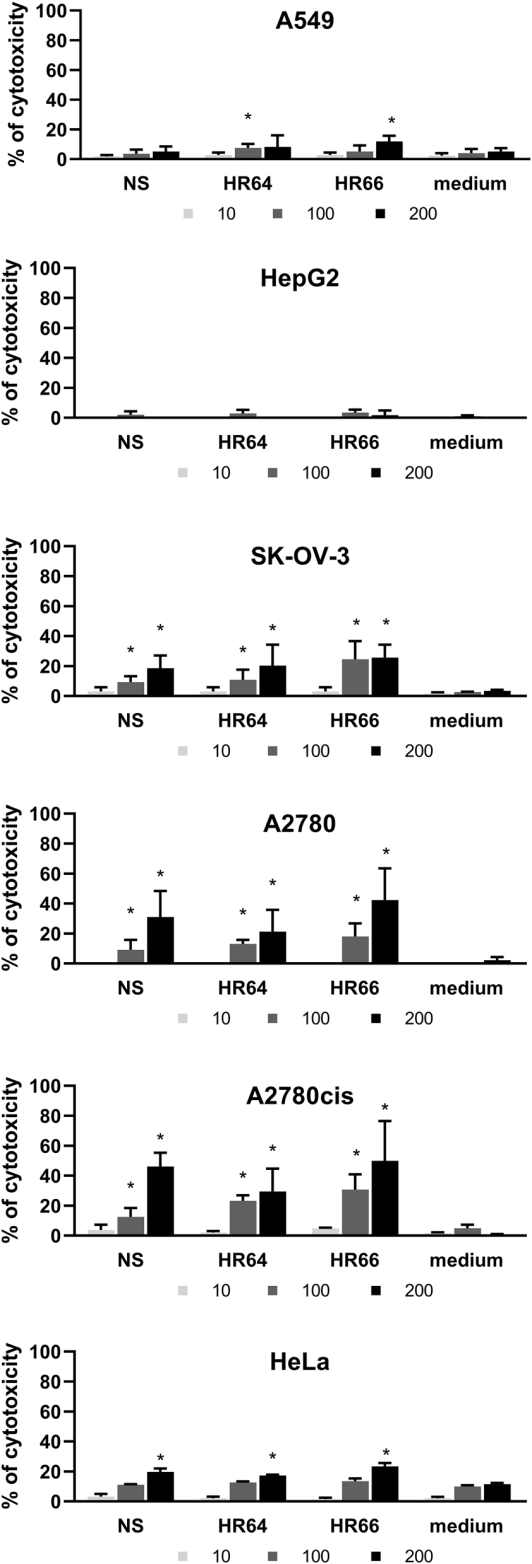
Cytotoxicity of halophilic archaea metabolites against cancer cell lines as determined by the MTT assay. A549, HepG2, SK-OV-3, A2780, A2780cis and HeLa cancer cell lines seeded at 5 × 10^4^ cells/well were treated with 10 (white bars), 100 (grey bars) or 200 µg/mL (black bars) of culture supernatant extracts of *Nnm. salaciae* (NS), *Hrd. rudnickae* 64^T^ (HR64) or *Hrd. rudnickae* 66 (HR66), or with fresh culture medium extract (medium) for 48 h at 37 °C and 5% CO_2_. Cytotoxicity was determined by using the MTT colorimetric assay and is expressed as mean percentages ± SD. n = 4; *p < 0.05 compared to medium

As presented in Fig. 2, none of the halophile samples affected cell metabolism at the concentration of 10 µg/mL, regardless of the cell line examined. The percentages of cytotoxicity did not exceed 10%, and these values were not significantly different from the control samples.

However, all halophilic samples exerted cytotoxic effects on ovarian carcinoma and on cervix adenocarcinoma cells (HeLa) at the doses of 100 and 200 µg/mL, albeit with different strengths. At 200 µg/mL, all extracts expressed 25% cytotoxicity on SK-OV-3 cells. The A2780 and A2780cis cells were also significantly sensitive to the cytotoxic effects of 100 and 200 µg/mL of all three halophilic samples. The metabolites of *Nnm. salaciae* and *Hrd. rudnickae* decreased the metabolism of A2780cis cells in a concentration-dependent manner by up to about 50%.

The metabolism of HeLa cells was also significantly affected by the *Nnm. salaciae* and *Hrd. rudnickae* metabolites, albeit at a low level and only at 200 µg/mL. Similarly, at high concentrations, the *Hrd. rudnickae* metabolites also affected the metabolism of A549 cells, but with a maximum toxicity of barely 10%. In contrast, HepG2 cells showed high resistance against all three archaeal samples, even at high concentrations. Since the spectrograms of control media containing 15% or 20% NaCl were identical to each other (Fig. 1A–C), Fig. 2 shows only the results of medium with 20% NaCl. The cytotoxic effect of 0.5% DMSO, corresponding to the concentration in the halophilic samples at 200 µg/mL, did not exceed 2% for any cell line tested (data not shown).

### xCELLigence RTCA assay

**Fig. 3:**
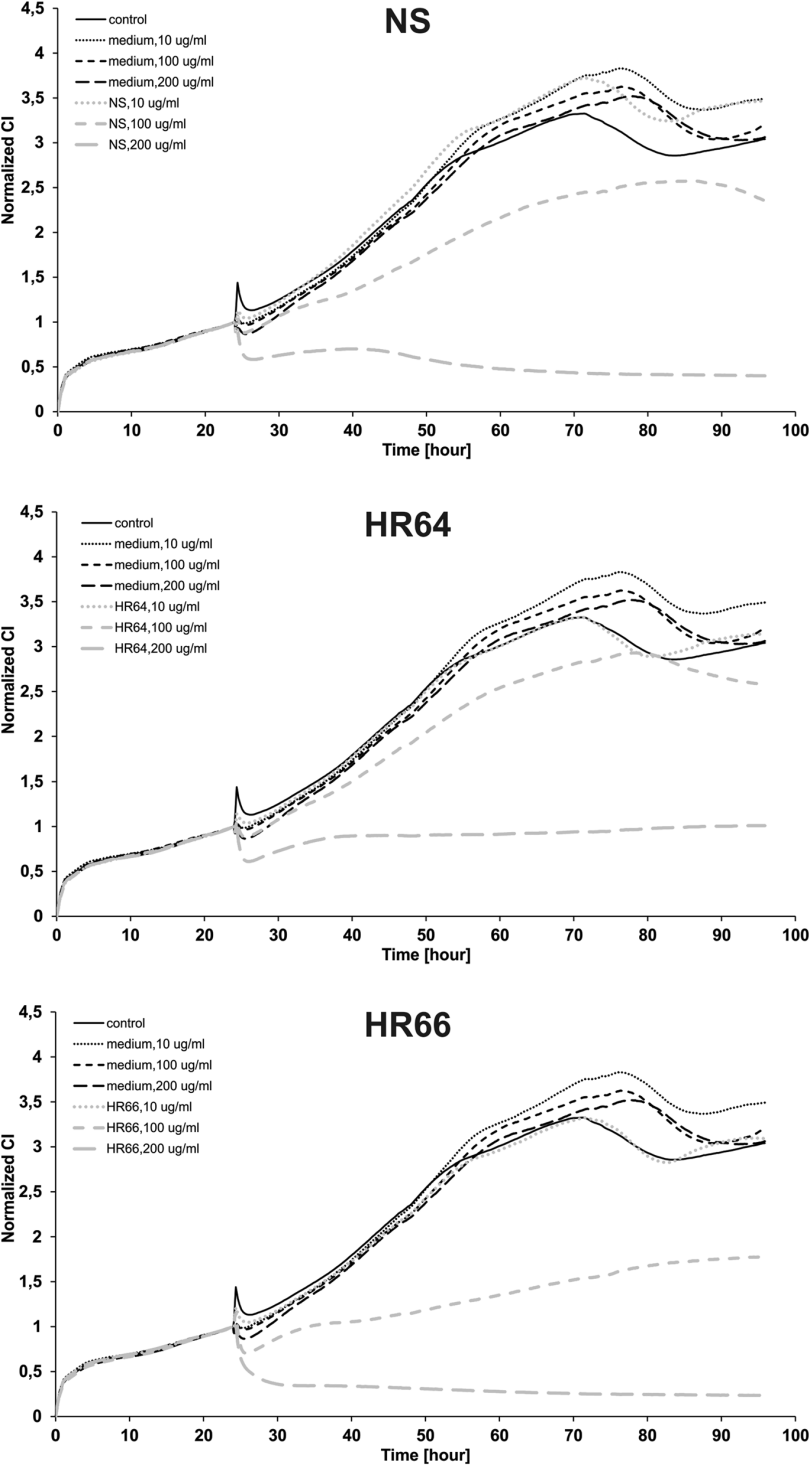
Real time impedance in A2780 cells treated with archaeal samples monitored using xCELLigence system. A2780 cells at 3 × 10^4^ cells/well were incubated for 24 h at 37 °C with 5% CO_2_ in an electronic 96-well plate and then treated with 10, 100 or 200 µg/mL of *Nnm. salaciae* (NS, upper panel), *Hrd. rudnickae* 64^T^ (HR64, middle panel) or *Hrd. rudnickae* 66 (HR66, lower panel) extract, with fresh culture medium extract or were left untreated, as indicated, for the following 72 h. During incubation, impedance was measured every 15 min in each individual well and automatically converted to Cell Index (CI) values. The graph presents characteristic curves of cells’ behaviour before and during treatment

Figure 3 shows an example of A2780 cell behaviour curves before and during treatment over time.

The initial increase in CI reflects the attachment of cells. This is followed by a period of proliferation. After the addition of *Nnm. salaciae* samples 24 h post-seeding, changes in the curves were noticed in a dose-dependent manner compared to the control samples. At the highest concentration of *Nnm. salaciae* metabolites the increase in CI was totally abolished, while it was not affected by 10 µg/mL up to 72 h of exposure. 100 µg/mL of the *Nnm. salaciae* sample expressed intermediate levels of cytotoxicity. As expected, medium alone did not significantly affect the increase in CI, regardless of the concentration tested. Similar results were obtained when the metabolites of the two *Hrd. rudnickae* strains were tested, although these two strains differed slightly from each other, as at 100 µg/mL the CI increase appeared to be more affected by the metabolites of *Hrd. rudnickae* 66 than of *Hrd. rudnickae* 64^T^.

**Fig. 4:**
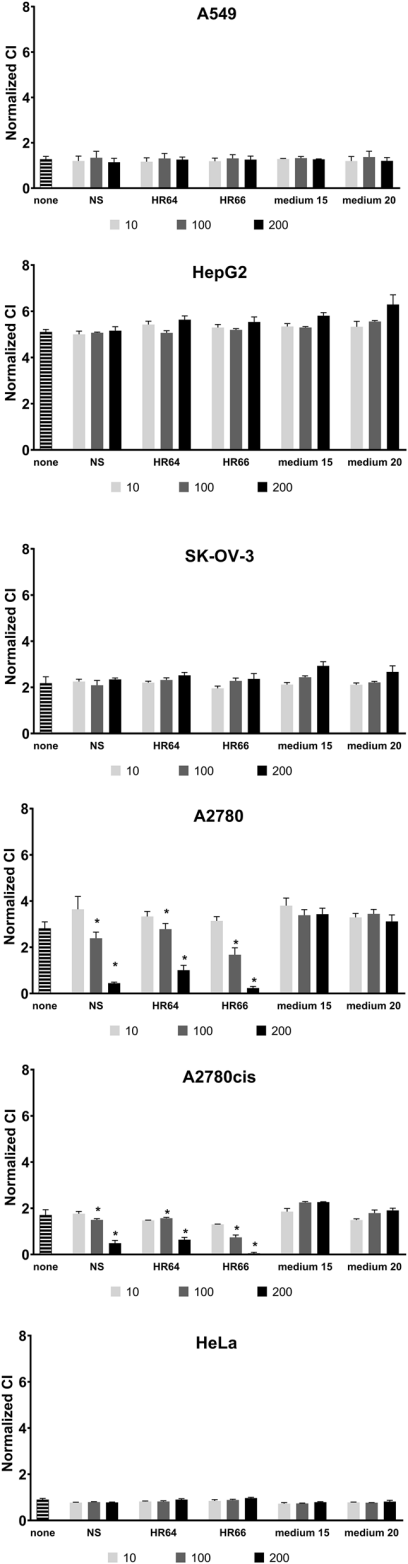
Normalized CI of cancer cell lines treated with halophilic archaea samples. A549, HepG2, SK-OV-3, A2780, A2780cis and HeLa cancer cell lines seeded at 3 × 10^4^ cells/well were incubated for 24 h in an electronic 96-well plate at 37 °C with 5% CO_2_ and then treated with 10 (white bars), 100 (grey bars) or 200 µg/mL (black bars) of extracts from *Nnm. salaciae* (NS), *Hrd. rudnickae* 64^T^ (HR64), *Hrd. rudnickae* 66 (HR66) or fresh culture medium extract (medium), or were left untreated (none, hatched bars) for the following 72 h. Impedance was measured every 15 min and converted to CI values. Data are presented as means ± SD at the final time point from 3 wells in 4 experiments. *p < 0.05 compared to medium

To compare the cytotoxicity of archaeal samples on the various cell lines, we measured the impedance at the final time point. No significant concentration-dependent changes in cell behaviour were observed for A549, HepG2, HeLa and SK-OV-3 cells treated with halophile samples, compared to the control medium (Fig. 4).

In contrast, substantial decreases in CI values for A2780 cells compared to controls were observed with the *Hrd. rudnickae* 64^T^, *Hrd. rudnickae* 66 and *Nnm. salaciae* samples in a dose-dependent manner. A2780cis cells were also found to be sensitive to the halophilic metabolites, and the *Hrd. rudnickae* 66 sample at a concentration of 200 µg/mL almost completely inhibited the increase of impedance in this cell line The CI values obtained for untreated cells or cells treated with a control medium containing 15% NaCl or 20% NaCl were similar, regardless of the cell line tested.

## Discussion

Halophilic microorganisms, such as *Halobacterium* sp. TM, *Halobacillus halophilus*, *Pseudomonas halophila*, *Halomonas* sp. HA1, *Halopenitus malekzadehii* M10418, *Halopenitus persicus* M10041 and *Haloferax mediterranei*, are known to produce various natural products, especially retinal proteins, hydrolytic enzymes and carotenoids with anti-cancer activity, associated with low levels of side effects [14–16]. However, the number of studies devoted to metabolites from halophilic archaea for potential cancer treatment is still very limited. Crude extracts from *Halomonas* sp. HA1, at concentrations of 20 and 40 µg/mL, induced apoptosis, inhibited proliferation and arrested the cell cycle at the G2/M phase of HepG2 cells [16]. Extracts of *Halovenus aranensis, Halorientalis persicus,* and *Halopenitus malekzadehii* reduced the viability of A549 cells, a non-small cell lung cancer cell line and MCF-7 and MDA-MB-468 cells, both breast cancer cell lines, but only at high concentrations of at least 400 µg/mL [14]. In contrast, the exopolysaccharide produced by *Halorubrum* sp. TBZ112 had no significant effect on the proliferation of human gastric cancer cell line (MKN-45) and on human dermal fibroblast cell line (HDF) [17].

Here we identified novel anti-cancer activities in culture media of two strains of *Hrd. rudnickae* [18] and one strain of *Nnm. salaciae* [19]. We chose these two strains because their genome sequences have been determined (accessible at GenBank assembly under the number GCA_900880625.1 and GCA_900110865.1, respectively), and we had already characterized their interaction with innate immune cells [20]*.* The metabolites of these strains were active on three ovarian cancer cell lines frequently used to study ovarian carcinoma: the cisplatin-sensitive ovarian tumor cell line A2780 and the corresponding cisplatin-resistant A2780cis cell line, as well as the cisplatin-resistant and highly invasive SK-OV-3 cell line, originating from ascites [21–23]. We also tested the archaeal samples on A549 cells, derived from non-small cell lung carcinoma, which have been largely used for cytotoxicity testing of new agents against lung cancer [24], as well as HeLa cells, derived from cervical carcinoma [25] and Hep2G cells derived from hepatocellular carcinoma, commonly used to study hepatotoxicity of compounds [26].

While metabolites from *Nnm. salaciae*, *Hrd. rudnickae* 64^T^ and *Hrd. rudnickae* 66, did not affect the metabolism and impedance increase of any of these cells at low concentrations of 10 µg/mL, significant cytotoxic and anti-proliferative activities of tested halophilic archaea metabolites were found at higher concentrations of 100 and 200 µg/mL, especially against ovarian cancer cells. Anti-cancer activities of crude halophilic extracts are considered promising when their IC_50_ is lower than 100 µg/mL [27], although anti-cancer substances isolated from natural sources, such as plants, fungi or bacteria, are generally active at higher than typical chemotherapeutic concentrations.

The specificity of cytotoxicity of halophilic supernatant metabolites for certain cell lines has been described previously. For instance, among various cell lines that were tested, including prostate cancer cells, breast cancer cells and lung cancer cell lines, only prostate cancer cells appeared to be sensitive to *Hbt. salinarum* extracts [14]. However, prior to this study, no halophilic archaea have been shown to produce metabolites that are specifically active against ovarian cancer cell lines.

Interestingly, by using two independent readouts for cytotoxicity, we identified different profiles between the ovarian cell lines. By using the MTT assay, which measures metabolic activities of cells [28], we found that all three ovarian cancer cell lines were sensitive to the metabolites of all three halophilic strains, although the cytotoxic effects seem to be somewhat stronger on A2780, and particularly on A2780cis cells than on SK-OV-3 cells. In contrast, when the xCELLigence RTCA assay was used, which measures cell growth and proliferation [13], SK-OV-3 cells appeared to be resistant to the archaeal metabolites, and only A2780 and A2780cis were affected by the metabolites, especially at the highest concentration tested. These observations indicate that, although at the concentrations tested, the archaeal metabolites might affect the metabolism of SK-OV-3 cells, this effect is not sufficient to have a significant effect on cell viability, growth and proliferation. Once identified, it will be of interest to determine whether higher concentrations of the purified metabolite(s) will also affect viability growth and proliferation of SK-OV-3 cells.

A surprising finding was that the culture supernatants of two strains of *Hrd. rudnickae* showed different ^1^H NMR spectra, although both strains exhibit 99.7% identity at the level of their 16S rRNA gene sequences, and their biochemical and physiological characteristics are also very similar [18]. However, this did not translate into significant differences in cytotoxicity between the two strains. While the diversity in peaks appeared to be larger for HR64^T^ than for HR66, some peaks were higher for HR66 than for HR64^T^. Although in this study we have not identified the active compounds, ^1^H NMR profiling shows that all three analyzed archaea secrete various peptides and aromatic metabolites, some of which appeared to be common to all three strains and may therefore be good candidates. Alternatively, the cytotoxic action of the three halophiles may rely on different mechanisms and therefore different molecules may be at play, which will await the identification of the active compounds in future studies. Preliminary whole-genome comparisons between the different strains did not reveal obvious metabolic pathways that could help to identify the anti-cancer metabolites.

## Conclusions

In conclusion, we demonstrate here that crude supernatant extracts of the three selected halophilic archaea express cytotoxicity specifically towards ovarian cancer cell lines in a dose-dependent fashion, while they show no, or very limited cytotoxicity towards other cell lines, such as non-small cell lung carcinoma, cervical carcinoma and hepatocellular carcinoma cell lines. To our knowledge, this is the first report of halophilic archaea producing metabolites with specific activity towards ovarian cancer cells, including cisplatin-resistant cells, which opens novel avenues for the treatment of these frequent and particularly difficult to manage cancers.

## Methods

### Halophilic archaea cultures

*Hrd. rudnickae* 64^T^ (DSM 29498^T^)*, Hrd. rudnickae* 66 (DSM 29499) and *Nnm. salaciae* (DSM 25055^T^) were kindly provided by Dr Luciana Albuquerque and Prof. Milton S. da Costa from the University of Coimbra, Portugal [18, 19]. Halophilic strains were cultivated in 100 mL of *Halobacteria* medium (HBM) (5 g/L yeast extract, 5 g/L casamino acids, 1 g/L Na-glutamate, 2 g/L KCl, 3 g/L Na_3_-citrate, 20 g/L MgSO_4_ × 7H_2_O,

36 mg/L FeCl_2_ × 4H_2_O, 360 ng/L MnCl_2_ × 4H_2_O) in 300 mL Erlenmeyer flasks. *Hrd. rudnickae *64^T^ and *Hrd. rudnickae* 66 were grown in halophilic medium with 20% of NaCl at 37 °C for 48 h, while *Nnm. salaciae* was grown in halophilic medium with 15% of NaCl at 45 °C for 48 h.

Growth of halophilic cultures was monitored by the optical density measurements at 600 nm (OD_600_), and colony-forming unit numbers were determined by growth on HBM containing 2% agar. Halophiles from 48 h cultures at logarithmic growth were harvested and centrifuged at 4 °C for 15 min at 4500×*g*. The culture supernatants were collected, transferred to the new tube and centrifuged again at 4 °C for 15 min at 4500×*g*. Two hundred mL of the final supernatant was used for organic metabolite extraction.

### Cell lines culture

The A2780 and A2780cis cell lines (human ovarian carcinoma) were purchased from ECACC General Cell Collection (Salisbury, UK), while the SK-OV-3 (human ovarian adenocarcinoma), A549 (human lung carcinoma), HeLa (human cervix adenocarcinoma) and HepG2 (human hepatocellular carcinoma) cell lines were purchased from ATCC (Manassas, VA, USA). All ovarian cancer cell lines were cultured in RPMI 1640 medium with 2 mM l-glutamine, 1 mM sodium pyruvate (Thermo Fisher Scientific, Foster City, CA, USA), 10% fetal bovine serum (FBS, Corning Life Sciences, Tewksbury, MA, USA) with 100 U/mL penicillin and 100 µg/mL streptomycin (Sigma-Aldrich, Darmstadt, Germany). The A549, HeLa and HepG2 cells were cultured in MEM medium (Sigma-Aldrich) supplemented with 10% FBS (Sigma-Aldrich), 2 mM l-glutamine (Sigma-Aldrich), 100 U/mL penicillin and 100 µg/mL streptomycin. All cell lines were cultured at 37 °C in a humidified 5% CO_2_ atmosphere and were passaged every 2–3 days using trypsin in 0.05% EDTA (Thermo Fisher Scientific) for 10–15 min at 37 °C with 5% CO_2_. After trypsinization cells were supplemented with appropriate culture medium and centrifuged at 200×*g* for 5 min at room temperature. One μM of cisplatin (Sigma-Aldrich) was added to the A2780cis cells every 2–3 passages in order to maintain drug resistance.

Before starting the experiment, the viability of cells was checked by using the trypan blue (Merck, Darmstadt, Germany) exclusion assay (> 95%).

### Preparation of archaeal metabolites

Organic metabolites of *Nnm. salaciae, Hrd. rudnickae* 64^T^ and *Hrd. rudnickae* 66 were extracted from the media after growth as described [15], with minor modifications. Briefly, the archaeal culture media (200 mL each) were extracted three times with 160 mL ethyl acetate (p.p.a. grade, Avantor Performance Materials, Gliwice, Poland). 50 mL of control media containing 15% NaCl or 20% NaCl were also extracted three times with 40 mL ethyl acetate. After separation, organic layers were combined and dried over MgSO_4_. Then, the drying agent MgSO_4_ was filtered, washed with ethyl acetate and the solvent was evaporated. Obtained residues were then dried under a vacuum for 72 h. Finally, dried media were weighed and dissolved in DMSO at a final concentration of 50 mg/mL.

### ^1^H NMR spectra

The spectra for ^1^H of the samples and controls were recorded on the Bruker Avance III 600 MHz spectrometer (Bruker Corporation, Billerica, MA, USA) equipped with a direct ATM probe at 600.26 MHz in DMSO-d_6_ (Cambridge Isotype Laboratories, MA, USA). As standard, the deuterated solvent was used.

### Cytotoxicity MTT colorimetric assay

A2780, A2780cis, SK-OV-3, HeLa, HepG2 and A549 cells in their appropriate culture medium were distributed into 96-well microplates (Nunc, Thermo Fisher Scientific) at a density of 5 × 10^4^ cells/well and incubated for 24 h at 37 °C with 5% CO_2_ to allow them to attach. Medium was then replaced by complete appropriate culture medium (see above), and the archaeal samples were added at concentrations of 10, 100 or 200 µg/mL. The cells were then incubated for 48 h at 37 °C with 5% CO_2_. Control cells were treated the same way in the absence of test samples. The stock solutions of extracts (50 mg/mL) were dissolved in culture medium before addition to cells, so that the final content of DMSO in samples did not exceed 0.5%. After incubation, culture supernatants were removed, and 100 µL of 2 mg/mL MTT was added to each well and incubated for 3 h at 37 °C with 5% CO_2_. MTT was then gently aspirated and formazan crystals were dissolved with 200 µL 2-propanol. Plates were shaken on the microplate shaker for 15 min, and absorbance was measured with the Multiskan RC plate reader (Labsystem, Helsinki, Finland) with a dual wavelength of 595 and 630 nm using Genesis Lite software. Each experiment was performed in triplicate and was repeated 3–4 times independently. Cell viability is presented as the percentage of cytotoxicity, calculated according to the formula.

$$\mathrm{Cytotoxicity \,\%}=\left(1-\left(\frac{\mathrm{optical \,density \,of \,sample}}{\mathrm{optical \,density \,of \,control}}\right)\right)\mathrm{x}100$$

### xCELLigence real time cell analysis

The xCELLigence real time cell analysis (RTCA) system (ACEA Bioscience, CA, USA) was used for measurements of cell viability. The cells were seeded at 3 × 10^4^ cells/well in culture medium (see above) into electronic 96-well plates (E-plates, ACEA Biosciences), and plates were placed on the RTCA station placed in the cell culture incubator at 37 °C with 5% CO_2_ to allow cells to proliferate until they reached confluency (approximately 24 h). Confluent monolayers were treated for 72 h with 10, 100 or 200 µg/mL of archaeal samples in triplicate or replaced with a fresh medium as controls. During incubation at 37 °C in 5% CO_2_, impedance from cells in each well was measured on the E-Plate and automatically converted to Cell Index (CI) values by the RTCA software. The impedance was measured every 30 min before archaeal sample addition and every 15 min after sample addition for the next 72 h.

### Statistical analysis

Statistical analyses were performed with the STATISTICA 12.0 PL program. Data are expressed as mean ± SD. Differences between samples were analyzed by the analysis of variance Kruskal–Wallis non-parametric test and Mann–Whitney U test. *p* values ≤ 0.05 were considered significant.

## Data Availability

All data generated or analyzed during this study are included in this published article. The raw data supporting the conclusions of this article will be made available by the authors, without undue reservation.
